# Age at Menopause and Risk of Developing Endometrial Cancer: A Meta-Analysis

**DOI:** 10.1155/2019/8584130

**Published:** 2019-05-29

**Authors:** Yanjun Wu, Wenjun Sun, Hui Liu, Dongfeng Zhang

**Affiliations:** Department of Epidemiology and Health Statistics, The College of Public Health of Qingdao University, Qingdao, Shandong Province, China

## Abstract

**Object:**

The association of age at menopause with endometrial cancer remains controversial. Therefore, we quantitatively summarized the evidence from observational studies with a meta-analysis.

**Methods:**

We searched PubMed, Web of Science, Embase, Medline, Chinese National Knowledge Infrastructure (CNKI), and Wan Fang Med online up to March 2019, and all eligible case-control and cohort studies were included in the study. Pooled relative risks (RRs) with 95% confidence intervals (CIs) were calculated using the random-effects model. The dose-response relationship was assessed by restricted cubic spline model. The heterogeneity among studies was evaluated by I^2^. Metaregression was used to explore the potential sources of between-study heterogeneity. Egger's test was used to estimate publication bias.

**Results:**

Eighteen articles including 957242 subjects with 4781 cases were included in the meta-analysis. The pooled RR (95%CI) of endometrial cancer for the highest versus the lowest age at menopause was 1.89 (95%CI: 1.58-2.26). For dose-response analysis, a nonlinear relationship was found between age at menopause and endometrial cancer, and the positive association became statistically significant when age at menopause was greater than 46.5 years old.

**Conclusions:**

This meta-analysis suggested that age at menopause was positively associated with endometrial cancer. For women whose menopausal age over 46.5 years old, the risk of endometrial cancer increased with the age at menopause.

## 1. Introduction

Endometrial cancer is the most common gynecological tumor of the female [1]. Globally, endometrial cancer causes approximately 5% of cancer cases and over 2% of cancer deaths in women [2, 3]. It ranks the fourth most common malignant tumor in the female in developed countries [4]. Present studies indicate that genetic factors, anthropometric factors, lifestyle factors (e.g., tobacco smoking, alcohol drinking, physical activity, and usual diet), and clinically relevant diseases (e.g., diabetes, polycystic ovary) are related to endometrial cancer risk [5–14]. Besides, many reproductive factors that increase continuous stimulation of estrogen can also result in a higher risk of endometrial cancer, such as parity [15], age at menarche [16], oral contraceptive use [17], and breastfeeding [18].

Menopause as the terminus of women reproductive life is generally defined as a stop of menstruation for a consecutive year [19]. The age at menopause is of great clinical and public health significance [20]. Considering women with a later menopausal age have higher hormone levels and longer lifetime exposure to estrogens [21], age at menopause may be associated with many diseases. Studies had found that menopausal age was related to the risk of breast cancer and liver cancer [22–25]. However, the association between age at menopause and endometrial cancer is still controversial.

In order to explore the association between age at menopause and the risk of endometrial cancer, a large number of epidemiologic studies have been conducted [4, 26–42]. Among these studies, ten studies suggested a significant association between later age at menopause and an increased risk of endometrial cancer [4, 26, 30, 32–36, 38, 41], but the effect size in different studies was various, whereas no significant association was found in the other eight studies [27–29, 31, 37, 39, 40, 42]. Therefore, we conducted a meta-analysis to quantitatively evaluate the association between age at menopause and the risk of endometrial cancer risk.

## 2. Materials and Methods

Preferred Reporting Items for Systematic reviews and Meta-Analyses (PRISMA) guidelines were adopted [43].

### 2.1. Search Strategy

**Figure 1:**
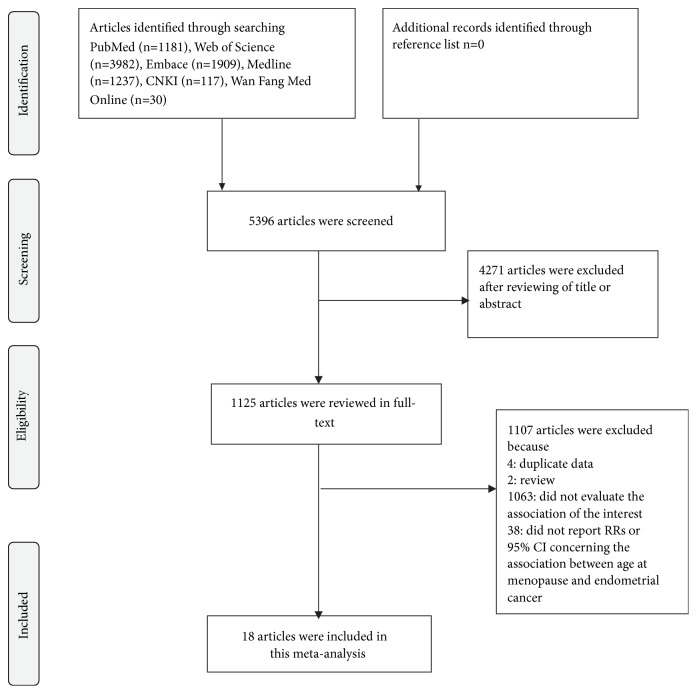
Flow chart of the selection of studies included in the meta-analysis.

 We used extended computer-based searches to obtain available studies published in English or Chinese from the databases of PubMed, Web of Science, Embase, Medline, Chinese National Knowledge Infrastructure (CNKI), and Wan Fang Med Online. The search terms used to search articles in this study were “age at menopause” (or “menopaus*∗* age” or “the age of postmenopause” or “age at climacteric” or “climacteric age” or “pausimenia age”) and “endometrial cancer” (or “endometrial neoplasm” or “endometrial carcinoma” or “carcinoma of endometrium”). Relevant references within included studies were also manually searched. The detailed steps of the literature selection were shown in Figure 1.

### 2.2. Inclusion Criteria

If the article met the following characteristics, it would be included in our meta-analysis. (1) An observational study (cohort or case-control) was published as an original article. (2) The exposure of interest was categorized age at menopause. (3) The outcome of interest was endometrial cancer. (4) There was reported effect size (relative risk (RR) or odds ratio (OR) or hazard ratio (HR) or incidence rate ratio (IRR)) and 95% confidence interval (CI) for the association between age at menopause and endometrial cancer. (5) We selected the most recent study if data from the same population were used in multiple articles.

All identified studies were searched and reviewed carefully by two investigators (Yanjun Wu and Wenjun Sun). If the two investigators had different views on the same article, it would be settled by discussing with the third investigator (Dongfeng Zhang).

## 3. Data Abstraction

From each eligible article, we extracted the first author's name, country in which the study was performed, publication year, the type of study design, the follow-up duration of cohort study, age range or mean age at baseline, the number of cases, and controls in case-control studies as well as the person-year of cases in cohort studies. We also abstracted the information about age at menopause, RRs (we presented all results as RR for simplicity) with their 95%CIs for each category of age at menopause, and adjustment factors in each study, menopausal type, the definition of postmenopausal status, and the source of case information.

## 4. Quality Assessment

The Newcastle-Ottawa Scale (NOS) [44] was used to assess the quality of case-control studies and cohort studies included in this study. The scale was composed of three parts (selection, comparability, and outcome), with a maximum score of 9 stars.

### 4.1. Statistical Analysis

The pooled measure was calculated as the inverse variance-weighted mean of the natural logarithm of RR with corresponding 95% CI to assess the strength of association between age at menopause and the risk of endometrial cancer. Heterogeneity among studies was assessed by *I*^2^ proposed by Higgins and Thompson [45]. Metaregression was performed to explore potential sources of between-study heterogeneity [46]. The influence analysis with one study removed at a time was carried out to evaluate whether a single study could affect the results significantly. Publication bias was evaluated using Egger's test and funnel plot [47].

For dose-response analysis, a two-stage random-effects dose-response meta-analysis [48] was performed. In the first stage, a restricted cubic spline model with three knots at the 25th, 50th, and 75th centiles of the levels of age at menopause was estimated using generalized least square regression [49], taking into account the correlation within each set of published RRs [50]. Then the study-specific estimates were combined using the restricted maximum likelihood method in a multivariate random-effects meta-analysis [51]. A *p* value for nonlinearity was calculated by testing the null hypothesis that the coefficient of the second spline is equal to 0. The details of the statistical method have been described elsewhere [52].

All statistical analyses were performed with StataV.15.0 (Stata Corp., College Station, TX, USA). All reported probabilities (*P* values) were two-sided, and* P* values less than 0.05 were considered statistically significant.

## 5. Results

### 5.1. Study Selection

According to the search terms mentioned in the section of Materials and methods, we identified 1181 articles from PubMed, 3982 articles from Web of Science, 1909 articles from Embase, 1237 articles from Medline, 117 articles from CNKI, and 30 articles from Wan Fang Med Online. We excluded 4271 articles by reviewing the title and abstract. In the step of full-text article reviewing, we further excluded 1107 articles. Among them, four articles had the same population, two articles were reviews, 1063 articles failed to evaluate the association between menopausal age and endometrial cancer, and 38 articles did not have RRs (95% CIs) concerning the interests. Ultimately, 18 articles [4, 26–42] were included in this meta-analysis. The detailed steps of the literature selection were presented in Figure 1.

### 5.2. Quality Assessment

After using NOS to assess the quality of the 18 articles included in this study, the mean Newcastle-Ottawa score was 7.8 (range from 6 to 9) for case-control studies and 8.1 (range from 7 to 9) for cohort studies. The detailed results of the quality assessment were summarized in Tables S1 and S2.

### 5.3. Study Characteristics

In the 18 articles, nine articles were case-control studies [27–29, 31–33, 36, 38, 41] and nine articles were cohort studies [4, 26, 30, 34, 35, 37, 39, 40, 42]. With regard to continent where the study was conducted, five studies were conducted in Asian [27, 29, 32, 34, 40], four studies [4, 26, 36, 41] in Europe, and nine studies in North America. The endometrial cancer cases of most studies [4, 26, 27, 30–33, 35, 38–42] were identified from registry records (such as cancer registry) and five studies [28, 29, 34, 36, 37] were from hospital medical records. Six studies [27, 28, 32, 35, 37, 39] included women only with natural menopause, five studies [31, 33, 34, 41, 42] included women with both natural menopause and surgical menopause, and other seven studies [4, 26, 29, 30, 36, 38, 40] did not have relevant information. Information about the definition of postmenopausal status and the detailed characteristics of the included studies were shown in Table 1.

### 5.4. Association between Age at Menopause and Endometrial Cancer

**Figure 2:**
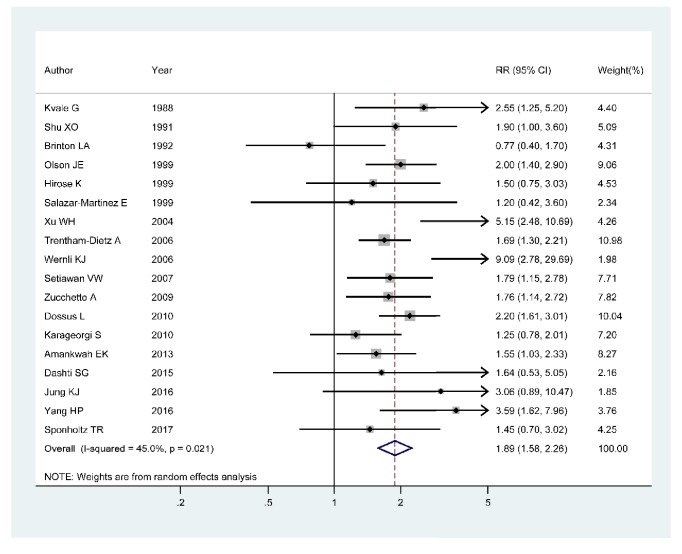
Forest plot of age at menopause and the risk of endometrial cancer. The size of gray box is positively proportional to the weight assigned to each study, and horizontal lines represent the 95% confidence intervals.

The association between age at menopause and endometrial cancer was evaluated in 18 articles [4, 26–42] with 957242 participants and 4781 cases. We could observe a statistically positive association between age at menopause and the risk of endometrial cancer in 10 articles [4, 26, 30, 32–36, 38, 41] of them, whereas the other eight studies [27–29, 31, 37, 39, 40, 42] showed no obvious association. The pooled RR of the risk of endometrial cancer for the highest versus the lowest age at menopause was 1.89 (95%CI 1.58–2.26; *I*^2^ = 45.0%, *P*_for  heterogeneity_=0.021) (Figure 2).

**Figure 3:**
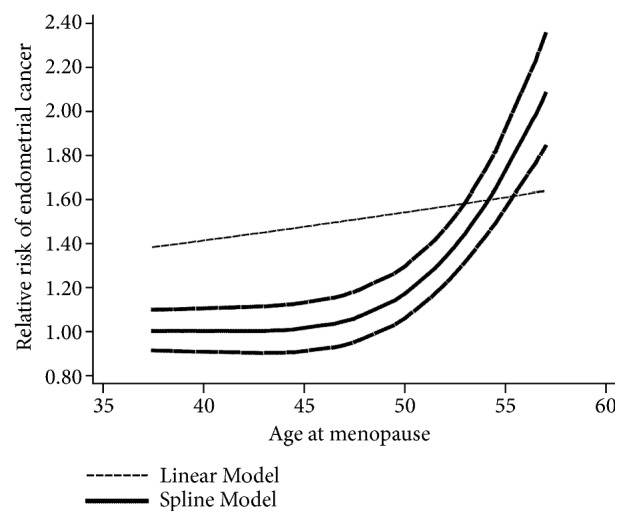
The dose-response analysis between age at menopause and the risk of endometrial cancer with restricted cubic splines in a multivariate random-effects dose-response model. The solid line and the long dash line represent the estimated relative risks and its 95% CIs. Short dash line represents the linear relationship.

In the dose-response analysis of 15 articles [4, 27–34, 36–38, 40–42], a nonlinear association between age at menopause and endometrial cancer was found (*P*_nonlinearity_ < 0.01). The positive association became significant when age at menopause was greater than 46.5 years old. The RRs (95% CIs) of endometrial cancer risk were 1.04 (1.03-1.06), 1.17 (1.14-1.20), 1.57 (1.45-1.71), and 2.08 (1.80-2.39) for 47, 50, 54, and 57 years old of age at menopause, respectively (Figure 3).

### 5.5. Subgroup Analysis

In the subgroup analysis by continent where the study was conducted, the pooled RRs (95%CIs) were 1.60 (1.37–1.86), 3.06 (1.64–5.72), and 2.19 (1.74–2.75) for North America, Asia, and Europe, respectively. In the subgroup analysis by study design, the pooled RRs (95%CIs) for case-control studies and cohort studies were 1.80 (1.36–2.38) and 1.98 (1.56–2.52), separately. In the subgroup analysis performed according to menopausal type, the pooled RRs (95%CIs) were 1.72 (1.09-2.71), 2.25 (1.31-3.85), and 1.94 (1.63-2.30) for studies among natural menopausal women, studies among both natural menopause and surgical menopause women, and studies without relevant information, respectively. In the subgroup analysis by reference group of menopausal age, the pooled RR (95%CI) was 2.06 (1.51-2.81) for studies that used age at menopause ≤45 (<45 or ≤ 45 or ≤40) as reference group and the pooled RR (95%CI) was 1.79 (1.52-2.10) for studies that used age at menopause ≤50 (<50 or ≤ 50 or ≤47) as the reference group. In the subgroup by the Newcastle-Ottawa score, the pooled RRs (95%CIs) were 1.67 (1.12-2.49) and 1.98 (1.66-2.37) for studies with a score of 6 or 7 and studies with a score of 8 or 9. The detailed results of subgroup analysis were summarized in Table 2.

### 5.6. Meta-Regression

Multivariable metaregression with the covariates of publication year (*p* = 0.727), study design (*p* = 0.623), mean age of study participants (*p* = 0.538), menopausal type (*p* = 0.621), reference group of menopausal age (*p* = 0.554), and the Newcastle-Ottawa score (*p* = 0.362) showed that these covariates had no significant impact on the heterogeneity. But continent where the study was conducted (*p *= 0.014) was found to have an influence on the between-study heterogeneity.

### 5.7. Influence Analysis and Publication Bias

**Figure 4:**
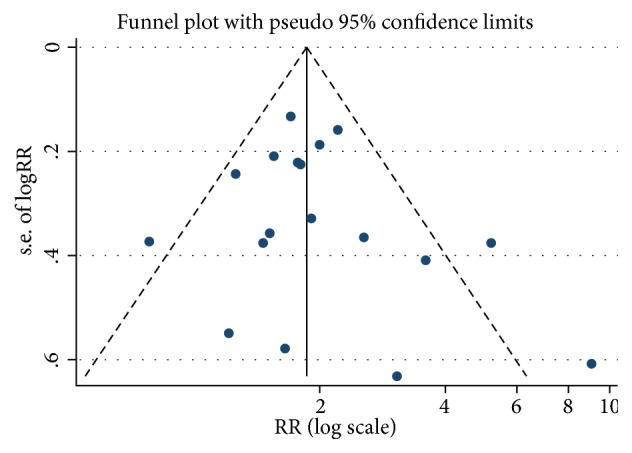
Funnel plot with pseudo 95% confidence limits for the analysis of age at menopause and risk of endometrial cancer (RR, relative risk).

No study had excessive influence on the pooled RR for the highest versus the lowest age at menopause in the influence analysis. No evidence of significant small-study effect for the analyses was found by the visual inspection of the funnel plot and Egger's test (*p*=0.373). The results of the funnel plot were shown in Figure 4.

## 6. Discussion

The average menopausal age for women varied among region [53]. Studies also showed that overweight, later age at menarche, and higher parity were associated with later menopausal age [54, 55]. In our study, we found that a later menopausal age was associated with an increased risk of endometrial cancer. In dose-analysis, a nonlinear relationship was found between age at menopause and endometrial cancer, and the positive association became statistically significant when age at menopause was greater than 46.5 years old. The positive association remained in subgroup analysis by continent, menopausal type, the reference group of menopausal age, and Newcastle-Ottawa score. Subgroup analysis especially by study design showed the positive association between age at menopause and endometrial cancer for cohort studies.

The mechanisms of higher endometrial cancer risk caused by later age at menopause are still equivocal. The hypothesis of “estrogen unopposed by progesterone” has been proposed as an important etiological factor [56, 57], which suggests that high levels of biological estrogens not counterbalanced by progesterone can result in a higher risk of endometrial cancer through increasing the mitotic activity of endometrial cells. Because the high-level female hormone can increase the probability of DNA-damaging (such as mitotic activity, DNA replication, and somatic mutations) and then become a fixed mutation [58, 59], people with a later menopausal age have higher hormone levels and longer time exposure to estrogens before menopause [21, 56]. In addition, later menopause might increase the risk of endometrial cancer by increasing the rate of spontaneous and environmentally induced mutations in endometrial stem cells [58]. Moreover, progesterone deficiency associated with anovulatory cycles is common in people having a later menopausal age, which may also contribute to endometrial cancer risk [60].

The issue of between-study heterogeneity should be paid particular attention in meta-analyses [61], and investigating the potential sources of between-study heterogeneity is necessary. The result of this meta-analysis showed moderate between-study heterogeneity in the analyses of age at menopause and risk of endometrial cancer. Multivariable metaregression with covariates of publication year, the continent where the study was conducted, study design, the mean age of study participants, menopausal type, reference group of menopausal age, and the Newcastle-Ottawa score was carried out to explore the potentially important source of heterogeneity. Among these covariates, just continent where the study was conducted was found to have a contribution to the between-study heterogeneity. After the subgroup analysis by continent, the positive association still remained in North America, Asia, and Europe and the pooled RRs were 1.60 (95% CI 1.37–1.86; *I*^2^= 0.0%  *P*_for  heterogeneity_= 0.516), 3.06 (95% CI 1.64–5.72; *I*^2^ = 63.5%  *P*_for  heterogeneity_= 0.027), and 2.19 (95% CI 1.74–2.75; *I*^2^ = 0.0%  *P*_for  heterogeneity_ = 0.453), respectively.

The study has many virtues. First, compared with the original individual study, our meta-analysis has a large number of included participants, which can make the results more precise and more reliable. Second, the quality of the included articles was relatively high with Newcastle-Ottawa score ranging from 6 to 9. Third, the results are still meaningful after subgroup analysis by continent, menopausal type, the reference group of menopausal age, and Newcastle-Ottawa score, and subgroup analysis by study design also showed the positive association between age at menopause and endometrial cancer in cohort studies. Finally, we conducted a dose-response analysis to explore the association between age at menopause and endometrial cancer quantitatively.

However, there are also several potential limitations in our study. First, the adjusted confounders are different among studies and the residual confounding could not be eliminated thoroughly. Second, the age range of participant is disparate in every study and years of follow-up in cohort studies are diverse. Finally, the definition of postmenopausal status and the stratification of age varied among studies, which might affect the result.

## 7. Conclusions

Results from this meta-analysis indicated that age at menopause was positively associated with the risk of endometrial cancer. When the menopausal age of women exceeded 46.5 years, the risk of endometrial cancer increased with her menopausal age. For these women, they should develop good lifestyles to reduce the risk of endometrial cancer. More well-designed prospective studies are needed to confirm the association in the future.

## Figures and Tables

**Table 1:** Characteristics of the included studies for age at menopause with risk of the endometrial cancer among postmenopausal women.

Author	Country (year)	Age range or mean age	Study design (years of follow up)	Age at menopause	Case (control or person-year)	Participants	RR(95%CI)	Adjustment for covariates	Menopausal type and definition of postmenopausal status	Source of case information
Kvale G [26]	Norway (1988)	27-69	Cohort (20)	≤45	16	62079	1	Age, residential type, parity	NA	Cases were identified by records from the Cancer Registry of Norway
				46-47	9		NA			
				48-49	20					
				50-51	21					
				52-53	15					
				≥54	15		2.55(1.25-5.20)			

Shu XO [27]	China (1991)	18-74 56.0/56.4	Case-control	≤45	29 (44)	404	1	Age, number of pregnancies, weight	①	Cases were identified by records from the Cancer Registry of Shanghai (1988-1990)
				46-48	25 (50)		1.00(0.50-2.00)			
				49-50	67 (56)		2.30(1.20-4.40)			
				≥51	73 (60)		1.90(1.00-3.60)			

Brinton LA [28]	America (1992)	20-74 59.2/58.0	Case-control	<45	39 (28)	504	1	Age, education, parity, recent weight, OC use, menopausal estrogen use	①	Cases were identified by medical records
				45-49	89 (69)		0.88(0.50-1.70)			
				50-54	133 (83)		0.99(0.50-1.80)			
				≥55	36 (27)		0.77(0.40-1.70)			

Hirose K [29]	Japan (1999)	30-80 56.6/48.5	Case-control	≤47	12 (1408)	7849	1	Age, BMI	NA	Cases were identified by medical records
				48-52	36 (4738)		0.88(0.45-1.69)			
				≥53	24 (1631)		1.50(0.75-3.03)			

Olson JE [30]	America (1999)	55-69	Cohort (10)	<45	41 (30853)	24848	1	Age	NA	Cases were identified by records from the State Health Registry of Iowa
				45-49	61 (56522)		0.80(0.60-1.20)			
				50-54	148 (106394)		1.10(0.80-1.50)			
				>55	82 (30987)		2.00(1.40-2.90)			

Salazar-Martinez E [31]	Mexico (1999)	57.1/54.6	Case-control	≤40	7 (32)	356	1	Age, anovulatory index, smoking, diabetes mellitus, hypertension, physical activity, BMI	②	Cases were from the gynecological oncology service and identified by biopsy-positive result.
				41-45	13 (61)		1.10(0.34-3.70)			
				46-50	16 (88)		1.00(0.34-3.30)			
				≥51	22 (117)		1.20(0.42-3.60)			

Xu WH [32]	China (2004)	30-69 55.2/55.1	Case-control	<45	31 (66)	1025	1	Age, education, OC use, alcohol drinking, any cancer history among first degree relatives, BMI, ever having had a live birth and ever having had an induce abortion, age at menarche	① Women were considered postmenopausal when they reported not having had any menses over the past 12 months	Cases were identified by records from the Cancer Registry of Shanghai (1997-2001)
				45-49	192 (247)		1.67(1.03-2.70)			
				50-54	220 (204)		2.38(1.46-3.87)			
				≥55	48 (17)		5.15(2.48-10.69)			

Trentham-Dietz A [33]	America (2006)	40-79 62.9/63.3	Case-control	<50	177 (716)	2464	1	Age	②	Cases were identified by records from Wisconsin Cancer Reporting System
				50-55	239 (866)		1.20(0.96-1.50)			
				>55	134 (332)		1.69(1.30-2.21)			

Wernli KJ [34]	China (2006)	NA	Cohort (9)	<45	4 (62227)	267400	1	Age, parity	② Women were considered postmenopausal when they reported not having had any menses over the past 6 months.	Cases were identified by medical record
				45-49	36 (310089)		2.09(0.56-7.75)			
				50-54	81 (394730)		3.73(1.03-13.47)			
				≥55	19 (38686)		9.09(2.78-29.69)			

Setiawan VW [35]	America (2007)	45-75 61.6	Cohort (7.3)	<45	NA	46933	1	Age, BMI, age at menarche, parity, OC use, smoking, diabetes, hypertension, family History of endometrial cancer, race	①	Cases were identified by records from Hawaii Tumor Registry
				45-49			1.27(0.88-1.84)			
				50-54			1.67(1.16-2.41)			
				≥55			1.79(1.15-2.78)			

Zucchetto A [36]	Italy (2009)	18-79 60/61	Case-control	<50	97 (252)	1077	1	Period of interview, BMI, age at menarche, parity, OC use	NA	Cases were identified by medical records.
				50-54	193 (358)		1.57(1.13-2.18)			
				≥55	64 (113)		1.76(1.14-2.72)			

Dossus L [4]	Denmark (2010)	NA	Cohort (8.7)	≤50	221 (535097)	302618	1	Age, BMI, physical activity, alcohol, diabetes, smoking, education	Women were considered postmenopausal when they reported not having had any menses over the past 12 months, or when they were older than 55 years.	Cases were identified by record linkage, health insurance records, cancer and pathology registries and active follow-up of study subjects.
				51-52	106 (170078)		1.32(1.04-1.68)			
				53-55	118 (156492)		1.49(1.18-1.89)			
				>55	53 (43212)		2.20(1.61-3.01)			
Karageorgi S [37]	America (2010)	30-50 41.8	Cohort (19.3)	<45	41 (71578)	121700	1	Age, parity, age at first birth, age at last birth, OC use, PMH use, BMI, smoking, diabetes, family history of endometrial cancer, age at menarche	①	Cases were identified by medical records.
				45-49	141 (310321)		0.82(0.57-1.18)			
				50-54	386 (521943)		1.22(0.81-1.85)			
				≥55	68 (77867)		1.25(0.78-2.01)			

Amankwah EK [38]	Canada (2013)	40-79 62.1/62.6	Case-control	<50	127 (297)	1103	1	Age, residential type, smoking, BMI, hypertension	Postmenopausal status was defined as age ≥60 years, or self-identified as postmenopausal with the last menstrual period ≥12 months, or 50 to 59 years and using menopausal hormones for ≥2 years.	Cases were identified by records from Alberta Cancer Registry
				50-54	176 (348)		1.23(0.92-1.64)			
				≥55	58 (97)		1.55(1.03-2.33)			

Dashti SG [39]	America (2015)	18-86 40	Cohort (15)	<50	5 (3357)	1128	1	Country, education, ascertainment, age at menarche, parity, PMH use	① Women were considered postmenopausal when they reported not having had any menses over the past 12 months	Cases were identified by records from Colon Cancer Family Registry
				≥50	14 (4375)		1.64(0.53-5.05)			

Jung KJ [40]	Korea (2016)	55.6	Cohort (12.1)	≤45	5	66466	1	Age, smoking, age at menarche, SBP, TC, HDL cholesterol, diabetes, exercise, exogenous hormone use, health insurance premium	Women were considered postmenopausal when they reported not having had any menses over the past 12 months	Cases were identified by records from National Health Insurance Service in Korea
				46-48	3		0.80(0.18-3.57)			
				49-51	12		2.68(0.82-8.78)			
				≥52	11		3.06(0.89-10.47)			

Yang HP [41]	Poland (2016)	20-74	Case-control	<45	33 (110)		1	Age, study site, age at menarche, oral contraceptive use, parity, LOC	②	Cases were identified by records from Polish Cancer Study
				45-49	111 (355)		1.44(0.83-2.48)			
				50-54	252 (579)		2.71(1.38-5.35)			
				≥55	114 (179)		3.59(1.62-7.96)			

Sponholtz TR [42]	America (2017)	21-69 36.6	Cohort (18)	<45	12 (22960)	47555	1	Age, study peroid, education, marital status, age at menarche, parity, menopausal status, OC use, estrogen-only female menopausal hormone use, estrogen plus progestin female menopausal hormone use, smoking status, BMI, physical activity	② Women were considered postmenopausal when they reported not having had any menses over the past 12 months, bilateral oophorectomy, or when they were ≥57 years of age and whose menopausal status was obscured due to hormone use.	Cases were identified by record linkage from state cancer registries
				45-49	38 (46155)		1.45(0.73-2.89)			
				50-54	64 (64053)		1.32(0.67-2.56)			
				≥55	28 (19161)		1.45(0.70-3.02)			

RR, relative risk; CI, confidence interval; NA, not available; BMI, body mass index; OC, oral contraceptive; PMH, postmenopausal hormone use; SBP, systolic blood pressure; TC, total cholesterol; HDL, high density lipoprotein; LOC, lifetime number of ovulatory cycles. ①, among women only with natural menopause; ②, among women with natural menopause and surgical menopause

**Table 2:** Summary of relative risks (RRs) for association of age at menopause with risk of the endometrial cancer.

Stratification	Number of studies	RR (95% CI)	*I* ^*2*^,%	*P*_for heterogeneity_
All studies	18	1.89 (1.58-2.26)	45.0%	0.021
Continent				
North America	9	1.60 (1.37-1.86)	0.0%	0.516
Asia	5	3.06 (1.64-5.72)	63.5%	0.027
Europe	4	2.19 (1.74-2.75)	0.0%	0.453
Study Design				
Case-control study	9	1.80 (1.36-2.38)	54.5%	0.024
Cohort study	9	1.98 (1.56-2.52)	35.4%	0.135
Menopausal type				
Natural menopause	6	1.72 (1.09-2.71)	67.1%	0.009
Natural menopause and surgical menopause	5	2.25 (1.31-3.85)	64.0%	0.025
NA	7	1.94 (1.63-2.30)	0.0%	0.729
Reference group of menopausal age				
≤45 (<45 or ≤45 or ≤40)	12	2.06 (1.51-2.81)	60.6%	0.003
≤50 (<50 or ≤50 or ≤47)	6	1.79 (1.52-2.10)	0.0%	0.761
The Newcastle-Ottawa score				
6 or 7	5	1.59 (1.18-2.15)	27.1%	0.241
8 or 9	13	2.05 (1.64-2.56)	50.8%	0.018

RR, relative risk; CI, confidence interval; BMI, body mass index; PMH, postmenopausal hormone

## Supplementary Materials

Supplementary Materials Table S1. Quality assessment of included case-control studies. Table S2. Quality assessment of included cohort studies.
