# Supplementary material for: Age at Menopause and Risk of Developing Endometrial Cancer: A Meta-Analysis
Source: Biomed Res Int. 2019 May 29;2019:8584130. doi: 10.1155/2019/8584130 (PMC6560333; doi:10.1155/2019/8584130)
Supplement: Supplementary Materials — Table S1. Quality assessment of included case-control studies. Table S2. Quality assessment of included cohort studies. [file 8584130.f1.zip › Table S1_BMRI_2766680.docx]

Table S1. Quality assessment of included case-control studies

|  | Author (year) | | | | | | | | |
| --- | --- | --- | --- | --- | --- | --- | --- | --- | --- |
| Selection | Shu XO[^41^](#_ENREF_41)  (1991) | Brinton LA[^40^](#_ENREF_40)  (1992) | Hirose K[^39^](#_ENREF_39)  (1999) | Salazar-Martinez E[^37^](#_ENREF_37)  (1999) | Xu WH[^36^](#_ENREF_36)  (2004) | Trentham-Dietz A[^35^](#_ENREF_35) (2006) | Zucchetto A[^32^](#_ENREF_32)  (2009) | Amankwah EK[^30^](#_ENREF_30)  (2013) | [Yang HP](https://www.ncbi.nlm.nih.gov/pubmed/?term=Yang%20HP%5BAuthor%5D&cauthor=true&cauthor_uid=27190045)[^27^](#_ENREF_27)  (2016) |
| 1. The case definition of endometrial cancer is adequate. | * | * | * | * | * | * | * | * | * |
| 1. Representativeness of the cases. | * | * | * | * | * | * | * | * | * |
| 1. Selection of controls. | * |  |  | * | * | * |  | * | * |
| 1. Definition of control is adequate. | * | * | * | * | * | * | * | * | * |
| Comparability^1^ |  |  |  |  |  |  |  |  |  |
| 1. Comparability of cases and controls on the basis of the design or analysis | ** | ** | ** | ** | ** | ** | * | ** | ** |
| Exposure |  |  |  |  |  |  |  |  |  |
| 1. Ascertainment of exposure | * | * | * | * | * | * | * | * | * |
| 1. Same method of ascertainment for cases and controls | * | * | * | * | * | * | * | * | * |
| 1. Non-Response rate | * |  |  | * |  |  |  |  |  |
| Overall quality score | 9 | 7 | 7 | 9 | 8 | 8 | 6 | 8 | 8 |

The quality of studies was assessed by the Newcastle-Ottawa quality assessment scale.

^1^A maximum of 2 stars could be awarded for this item. Studies that controlled for age or gender received 1 star, whereas studies that controlled for other important confounders (such as parity, BMI, or age at menarche) received 1 additional star.

One star represents a score of 1, and a study can be awarded a maximum score of 9 (9 stars) in total.
