# Supplementary material for: Age at Menopause and Risk of Developing Endometrial Cancer: A Meta-Analysis
Source: Biomed Res Int. 2019 May 29;2019:8584130. doi: 10.1155/2019/8584130 (PMC6560333; doi:10.1155/2019/8584130)
Supplement: Supplementary Materials — Table S1. Quality assessment of included case-control studies. Table S2. Quality assessment of included cohort studies. [file 8584130.f1.zip › Table S2_BMRI_2766681.docx]

Table S2. Quality assessment of included cohort studies

|  | Author (year) | | | | | | | | |
| --- | --- | --- | --- | --- | --- | --- | --- | --- | --- |
|  | Kvale G[^42^](#_ENREF_42)  (1998) | Olson JE[^38^](#_ENREF_38)  (1999) | Wernli KJ[^34^](#_ENREF_34)  (2006) | Setiawan VW[^33^](#_ENREF_33)  (2007) | Dossus L[^4^](#_ENREF_4)  (2010) | Karageorgi S[^31^](#_ENREF_31)  (2010) | Dashti SG[^29^](#_ENREF_29)  (2015) | Jung KJ[^28^](#_ENREF_28)  (2016) | Sponholtz TR[^26^](#_ENREF_26)  (2017) |
| Selection |  |  |  |  |  |  |  |  |  |
| 1. Representativeness of the exposed cohort | * | * | * | * | * | * | * | * | * |
| 1. Selection of the non exposed cohort | * | * | * | * | * | * | * | * | * |
| 1. Ascertainment of exposure | * | * | * | * | * | * | * | * | * |
| 1. Outcome of interest not present at start of study | * | * | * | * | * | * | * | * | * |
| Comparability |  |  |  |  |  |  |  |  |  |
| 1. Comparability of cohorts on the bias of the design or analysis | ** | * | ** | ** | ** | ** | * | ** | ** |
| Outcome |  |  |  |  |  |  |  |  |  |
| 1. Assessment of outcome | * | * | * | * | * | * | * | * | * |
| 1. Follow-up long enough for outcomes to occur | * | * | * | * | * | * | * | * | * |
| 1. Adequacy of follow-up of cohorts | * |  | * |  |  |  |  |  | * |
| Overall quality score | 9 | 7 | 9 | 8 | 8 | 8 | 7 | 8 | 9 |

The quality of studies was assessed by the Newcastle-Ottawa quality assessment scale.

^1^A maximum of 2 stars could be awarded for this item. Studies that controlled for age or gender received 1 star, whereas studies that controlled for other important confounders (such as parity, BMI, or age at menarche) received 1 additional star.

^2^A cohort study with a follow-up time ＞3 years was assigned 1 star.

^3^A cohort study with a follow-up rate ＞70% was assigned 1 star.

One star represents a score of 1, and a study can be awarded a maximum score of 9 (9 stars) in total.
